# Dress

**Published:** 1889-05

**Authors:** 


### DRESS.

Nothing gives greater width to the bust than a short mantle, which gives extra width to the shoulder—a thing which should always be avoided, for a woman's shoulder to be beautiful should be rounded and drooping. not square and high, as we have seen too frequently of late. The beauty of a woman's bust depends in great measure on this curve between the neck and arm, and, therefore, no fashion should ever be adopted which destroys this beauty which nature has given to woman, none which may tend to make a woman's shoulders resemble those of a Life Guardsman. Look at all the statues of antiquity, fashion-makers! and tell me if you see *one* perfect woman's form with high, square shoulders. Why, then, should fashion-makers impose *their* whims on women in general, in order to please, perhaps, some ill-shaped wealthy customer or high-born personage? When such is the case, every woman should revolt and strike against all fashions that distort nature for the benefit of some deformed unit.

